# Deucravacitinib Treatment for Psoriasis Complicated by Myelodysplastic Syndrome Without Exacerbation of the Underlying Hematologic Disorder: A Case Report

**DOI:** 10.7759/cureus.84075

**Published:** 2025-05-14

**Authors:** Yoshihito Mima, Masako Yamamoto, Ken Iozumi

**Affiliations:** 1 Department of Dermatology, Tokyo Metropolitan Police Hospital, Tokyo, JPN

**Keywords:** deucravacitinib, jak-stat, myelodysplastic syndrome, psoriasis, tyk2

## Abstract

Psoriasis is a chronic, immune-mediated inflammatory skin disease associated with systemic comorbidities such as cardiovascular disease and autoimmune disorders. Deucravacitinib, a selective oral tyrosine kinase 2 (TYK2) inhibitor, has demonstrated strong efficacy and safety in the treatment of psoriasis. However, its use in patients with hematologic malignancies, such as myelodysplastic syndrome (MDS), has not been thoroughly investigated. We report the case of a 73-year-old man with psoriasis vulgaris complicated by MDS and chronic kidney disease (CKD). The patient had a long-standing history of psoriasis managed with topical therapies but experienced an acute flare, presenting with erythema, scaling, and pustules involving over 40% of the body surface area (Psoriasis Area and Severity Index (PASI) score: 28.8). Given his underlying MDS and the need for a safe therapeutic option, deucravacitinib (6 mg once daily) was initiated. Over eight months of treatment, the patient's skin lesions markedly improved, achieving a PASI 75 response with a final PASI score of 4.6. No adverse effects or signs of MDS progression were observed. Moreover, his anemia remained well controlled without the need for monthly darbepoetin alfa injections. This case highlights the potential of deucravacitinib as a safe and effective treatment option for psoriasis patients with concurrent MDS. Given the emerging understanding of TYK2 as an oncogenic driver in myeloproliferative diseases, further studies are warranted to clarify the therapeutic implications of TYK2 inhibition in this patient population.

## Introduction

Psoriasis is a chronic, immune-mediated inflammatory skin disease that significantly impairs patients' quality of life [1,2]. Clinically, it typically presents as sharply demarcated erythematous plaques covered with silvery-white lamellar scales, commonly affecting areas such as the scalp, gluteal region, elbows, and knees [3]. Psoriasis has a complex pathogenesis and is associated with a wide range of systemic comorbidities, including cardiovascular disease, metabolic syndrome, obesity, diabetes, hypertension, autoimmune disorders, malignancies, inflammatory bowel disease, nonalcoholic fatty liver disease, and depression [4].

In psoriasis, T helper (Th)1 and Th17 cells play critical roles in disease pathogenesis. Interleukin (IL)-12 is essential for the differentiation and proliferation of Th1 cells, leading to the production of interferon-γ and tumor necrosis factor (TNF)-α. IL-23, on the other hand, is central to the survival and expansion of Th17 cells [5]. Consequently, IL-12 and IL-23 are considered the key pathogenic mediators in psoriasis [5]. Activated Th1 and Th17 cells release cytokines such as IL-17A/F, IL-22, and TNF-α, which drive excessive keratinocyte proliferation and inflammation in both the epidermis and dermis [5-7]. In addition to many injectable biologics targeting IL-17 or IL-23 pathways, deucravacitinib has emerged as a novel oral therapy that suppresses keratinocyte hyperproliferation and inflammation through Th17 cell modulation [8,9].

Deucravacitinib is a selective, oral tyrosine kinase 2 (TYK2) inhibitor [9]. TYK2, a member of the Janus kinase (JAK) family, mediates the intracellular signaling of IL-12, IL-23, and type I interferons [10]. By selectively inhibiting TYK2, deucravacitinib disrupts these cytokine-signaling pathways, effectively controlling keratinocyte hyperproliferation and inflammation in psoriasis [9,10]. The clinical efficacy of deucravacitinib has been demonstrated in two large phase III trials comparing it to both placebo and apremilast in patients with psoriasis [9]. More than 80% of patients achieved a Psoriasis Area and Severity Index (PASI) 75 response, defined as a ≥75% improvement in skin symptoms, within six months [9]. Common adverse events associated with deucravacitinib include nasopharyngitis, upper respiratory tract infections, headache, diarrhea, and nausea. Unlike conventional JAK inhibitors, deucravacitinib has demonstrated a favorable safety profile, with low incidences of serious infections, thromboembolic events, and clinically significant laboratory abnormalities. The overall frequency of adverse events was comparable to that observed in the placebo group [9]. Given its demonstrated efficacy and safety, deucravacitinib is now considered a viable systemic treatment option for psoriasis, alongside IL-17 and IL-23 inhibitors. Compared to these biologic agents, deucravacitinib may be somewhat less potent in terms of efficacy and speed of onset, but it offers the advantage of a more favorable safety profile [9,10].

Myelodysplastic syndrome (MDS) was first established as a distinct disease entity by the French-American-British (FAB) Group in 1982. MDS is a hematologic disorder characterized by cytopenia, particularly anemia, with typically hypercellular bone marrow. Dysplastic hematopoiesis involving one or more hematopoietic lineages is observed [11].

To date, there have been very few reports of psoriasis with hematologic disorders such as MDS treated with deucravacitinib, and little discussion regarding the relationship between MDS and TYK2. Herein, we report a case of psoriasis with concurrent MDS successfully treated with deucravacitinib, resulting in significant improvement of the psoriatic skin lesions without exacerbation of the underlying MDS.

## Case presentation

**Figure 1:**
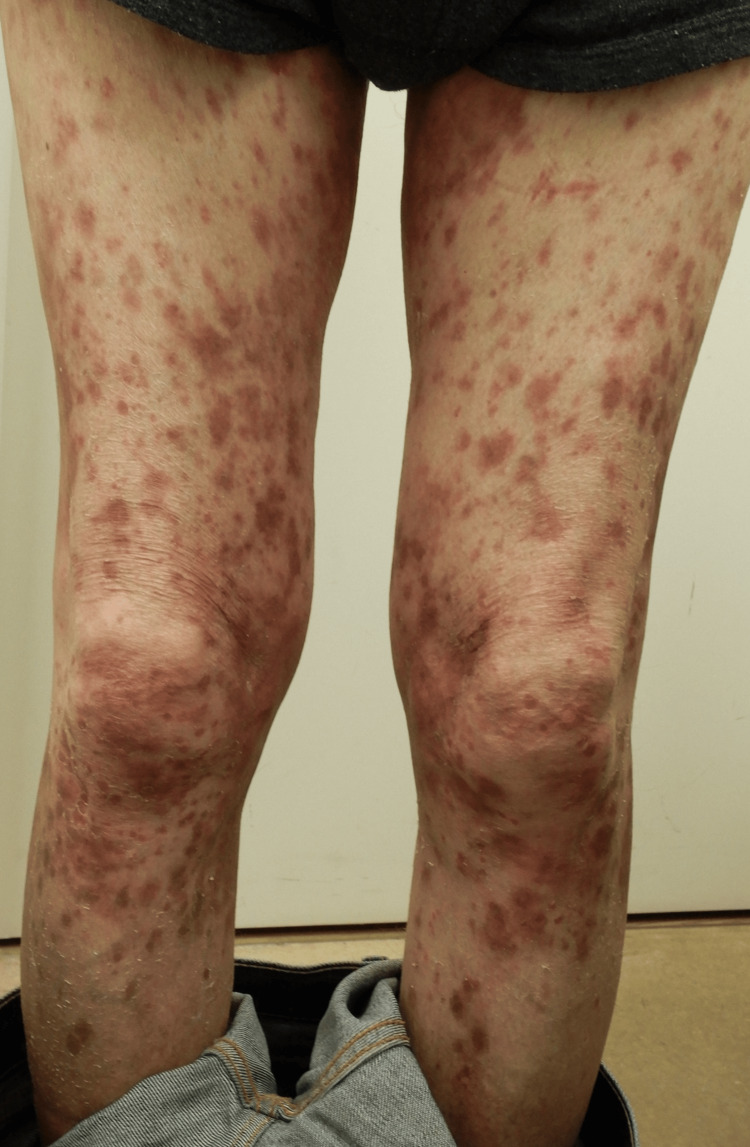
Widespread erythematous lesions with scaling were diffusely distributed across the trunk and extremities

**Figure 2:**
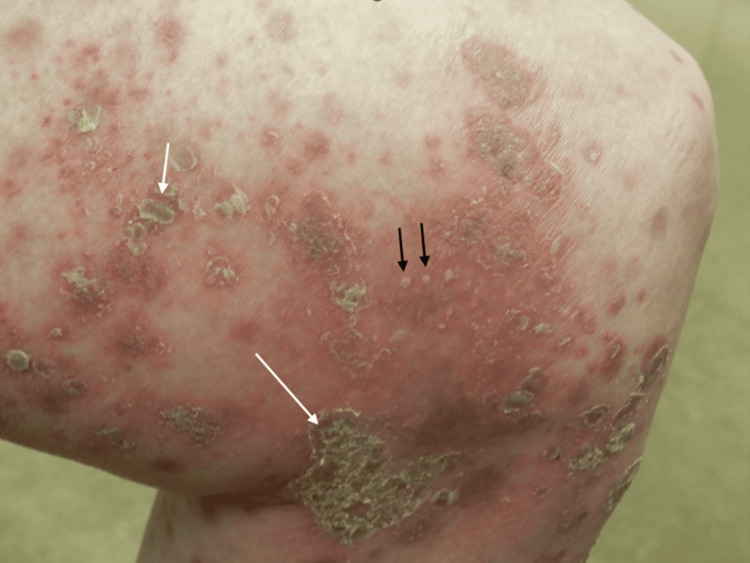
The erythematous lesions with scaling were partially accompanied by crusts (white arrows) and small pustules (black arrows)

A 73-year-old man with a more than 10-year history of psoriasis had been managed with topical calcipotriol hydrate and betamethasone dipropionate. His past medical history included chronic kidney disease (CKD) and MDS. The CKD was being managed conservatively, as there was no proteinuria, and his blood pressure remained stable. Regarding MDS, bone marrow aspiration performed three years earlier had confirmed a low-risk classification, and the only hematologic abnormality was mild anemia, for which he received monthly injections of darbepoetin alfa, an erythropoiesis-stimulating agent. The patient was referred to our department due to an acute exacerbation of his psoriatic skin lesions, which had become difficult to control with the existing topical therapies. Physical examination revealed widespread erythematous lesions with scaling and crusting over the trunk and extremities, with scattered pustules noted in some areas (Figure 1 and Figure 2).

The skin lesions involved more than 40% of the body surface area, and the Psoriasis Area and Severity Index (PASI) score was 28.8. No systemic symptoms, such as joint tenderness or low-grade fever, were present. Laboratory tests showed elevated C-reactive protein (1.5 mg/dL) and lactate dehydrogenase (284 IU/L) levels. Additional laboratory findings are summarized in Table 1.

**Table 1:** Laboratory examination result RR: reference range, AST: aspartate aminotransferase, ALT: alanine aminotransferase, Alk Phos: alkaline phosphatase, LDH: lactate dehydrogenase, CRP: C-reactive protein, WBC: white blood cell, Hb: hemoglobin, Plt: platelet

Variable	Patient value	RR, adults
AST	18 U/L	11-33 U/L
ALT	23 U/L	6-37 U/L
Alk Phos	101 U/L	35-104 U/L
Albumin	3.5 g/dL	3.8-5.0 g/dL
Total protein	6.3 g/dL	6.1-8.2 g/dL
Total bilirubin	0.4 mg/dL	0.2-1.2 mg/dL
LDH	284 U/L	135-214 U/L
CRP	1.5 mg/dL	<0.3 mg/dL
WBC	6,300/μL	3,500-8,500/μL
Hb	11.9 g/dL	13.5-17.0 g/dL
Plt	181×10^3^/μL	870-1,700 mg/dL

**Figure 3:**
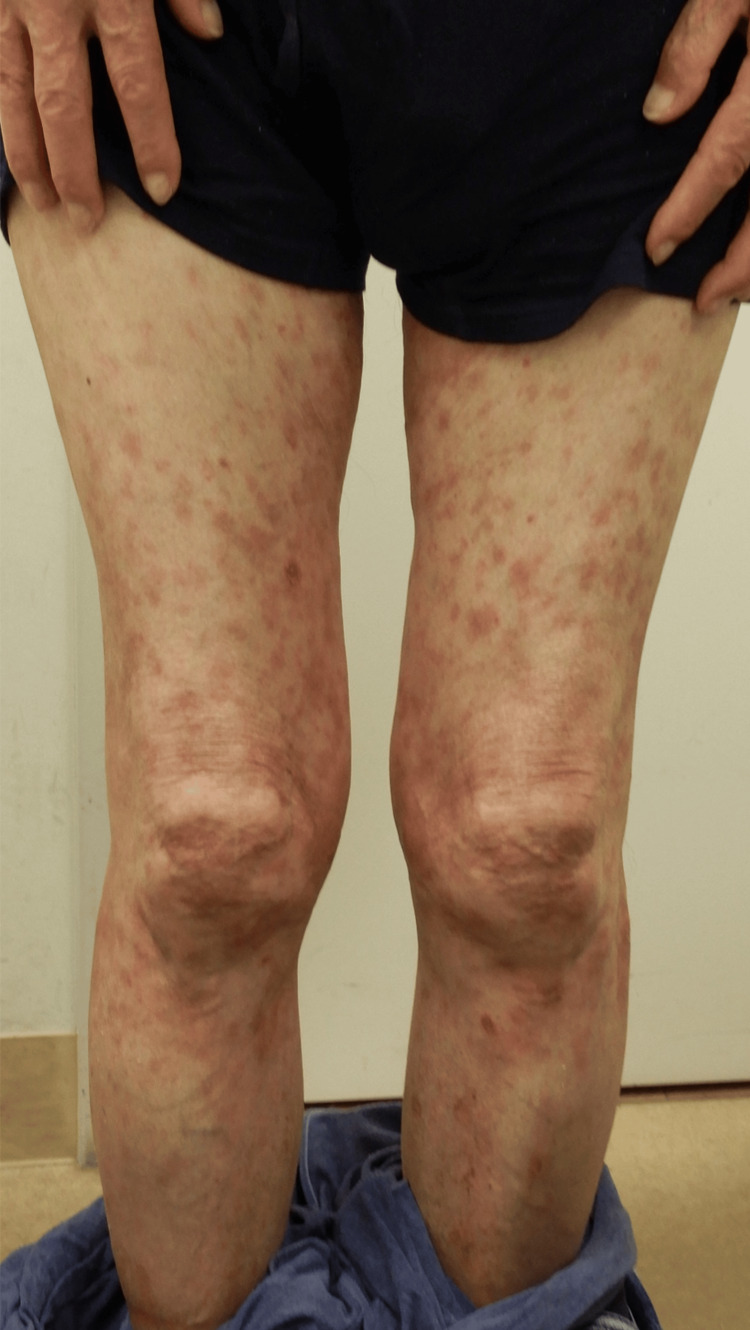
The erythematous lesions with scaling on the trunk and extremities had mostly evolved into areas of post-inflammatory hyperpigmentation after eight months of deucravacitinib treatment

Bacterial cultures from pustules were negative. Given the patient's long-standing history of psoriasis and the current flare, we diagnosed a transition from psoriasis vulgaris toward pustular psoriasis. After discussing treatment options with the patient, including IL-17 inhibitors, IL-23 inhibitors, and deucravacitinib, he expressed a preference to begin with the medication associated with the lowest risk of adverse effects, given his underlying MDS. Based on this consideration, treatment was initiated with deucravacitinib (6 mg/day), a selective TYK2 inhibitor with a favorable safety profile. The skin lesions gradually improved, and after eight months of treatment, his PASI score had decreased to 4.6 (Figure 3).

No adverse effects attributable to deucravacitinib were observed, and no progression of MDS was noted. Furthermore, prior to the initiation of deucravacitinib, the patient's anemia was managed with monthly darbepoetin alfa injections, which maintained hemoglobin levels between 10 and 12 g/dL. However, following the initiation of deucravacitinib, hemoglobin levels remained stable within the same range (10-12 g/dL) without the need for continued darbepoetin alfa administration. He continues to receive deucravacitinib with ongoing clinical follow-up.

## Discussion

In a clinical trial conducted in Japan evaluating deucravacitinib for psoriasis, approximately 80% of patients achieved PASI 75 and about 50% achieved PASI 90 at 24 weeks, demonstrating its efficacy [12]. In our case as well, the patient's PASI score improved from 27.6 to 4.6 over eight months, achieving PASI 75. However, considering that the proportion of patients achieving PASI 90 did not increase beyond 24 weeks in clinical trial data, it is unlikely that further significant improvement in PASI will occur beyond this point in our case. Given the patient's underlying conditions, including MDS and chronic kidney disease, which place him at higher risk for complications such as infections, any future consideration of switching to biologic therapy will need to be approached with great caution.

A key feature of the present case is the administration of deucravacitinib to a psoriasis patient with underlying myelodysplastic syndrome (MDS), a hematologic malignancy. In clinical trials, deucravacitinib has not been administered to patients with hematologic malignancies [9,11], and to the best of our knowledge, only one prior case report has described such use [13]. Huang et al. reported a case in which a TYK2 inhibitor was used to treat reactive granulomatous dermatitis (RGD) associated with MDS [13]. In their case, similar to ours, the patient had stable cytopenias under observation without active treatment, and no progression of MDS was observed following the administration of deucravacitinib.

The activation of the JAK-STAT signaling pathway has been implicated in the pathogenesis of myeloproliferative disorders. Specifically, the somatic V617F mutation in the pseudokinase domain of JAK2 is recognized as a major driver mutation in many chronic myeloproliferative neoplasms. Functional studies investigating the homologous V678F mutation in TYK2 demonstrated that V678F TYK2 enhances STAT3 and STAT5 transcriptional activity, leading to autonomous cell proliferation [14]. Furthermore, MDS and acute myeloid leukemia (AML) are strongly associated with sustained, dysregulated activation of STAT3/5 [15]. Thus, in addition to JAK2 V617F, TYK2 V678F mutations may also contribute to the development of myeloproliferative disorders, including MDS [14,15].

Previous studies have reported TYK2 mutations in hematologic malignancies, particularly in myeloproliferative disorders such as acute lymphoblastic leukemia. Constitutive activation of mutant TYK2 results in the phosphorylation of STAT1/3/5, promoting continuous cell proliferation. Knockdown of TYK2 fusion proteins has been shown to reduce STAT phosphorylation and inhibit cell growth, supporting the role of TYK2 as an oncogenic driver kinase. Furthermore, pharmacological inhibition of TYK2 suppressed phosphorylation of STAT1/3/5 and attenuated persistent cell proliferation [16]. In MDS, next-generation sequencing of bone marrow cells has identified TYK2 mutations in some cases [17].

Taken together, these findings suggest that TYK2 may function as a proliferative oncogenic driver in myeloproliferative disorders, including MDS. Therefore, TYK2 inhibition by deucravacitinib is unlikely to exacerbate such conditions. On the contrary, deucravacitinib may potentially exert anti-proliferative effects in disorders like MDS. However, the role of TYK2 as an oncogenic driver has only recently gained attention, and both basic research and clinical data exploring its association with MDS and related hematologic conditions remain limited. In the present case, hemoglobin levels remained stable after the discontinuation of erythropoiesis-stimulating agents following the initiation of deucravacitinib. Nonetheless, the possibility that the erythropoietin therapy had been ineffective to begin with, or that this outcome was coincidental, cannot be excluded. Therefore, the extent to which deucravacitinib may have influenced anemia or MDS in this patient remains unclear. Furthermore, this report is based on a single case, which represents a key limitation. Additionally, our case only reflects short- to mid-term outcomes, with deucravacitinib administered for eight months. Therefore, long-term evaluation over several years is needed to assess the drug's potential hematologic effects. To gain a deeper understanding of the potential relationship between deucravacitinib and MDS, further accumulation and careful evaluation of cases involving patients with coexisting psoriasis and MDS are essential.

## Conclusions

The use of biologic agents and TYK2 inhibitors in patients with psoriasis complicated by hematologic malignancies such as myelodysplastic syndrome (MDS) has been scarcely investigated to date. Therefore, this case represents a valuable contribution, suggesting that deucravacitinib may serve as a safe and effective therapeutic option for patients with coexisting psoriasis and MDS. Recent studies have highlighted TYK2 as a potential oncogenic driver in myeloproliferative disorders, suggesting that TYK2 inhibition is unlikely to exacerbate diseases such as MDS. However, our understanding of TYK2's role as an oncogenic kinase is still in its early stages, and both basic research and clinical data on its relevance to MDS and other myeloproliferative neoplasms remain limited. Further case accumulation and ongoing investigation will be essential to elucidate this potential association.
